# A novel Mediterranean diet index from Lebanon: comparison with Europe

**DOI:** 10.1007/s00394-014-0801-1

**Published:** 2014-11-20

**Authors:** Farah Naja, Nahla Hwalla, Leila Itani, Shirine Baalbaki, Abla Sibai, Lara Nasreddine

**Affiliations:** 1 Department of Nutrition and Food Sciences, Faculty of Agriculture and Food Sciences, American University of Beirut, Riad El-Solh, P.O. Box 11-0236, Beirut, 1107 2020 Lebanon; 2 Department of Nutrition and Dietetics, Faculty of Health Sciences, Beirut Arab University, Riad El-Solh, Beirut, 1107 2809 Lebanon; 3 Department of Epidemiology and Population Health, Faculty of Health Sciences, American University of Beirut, Riad El-Solh, Beirut, 1107 2020 Lebanon

**Keywords:** Mediterranean diet, Adherence, Middle East, Lebanon, Europe

## Abstract

**Purpose:**

To propose an index for assessing adherence to a Middle Eastern version of the Mediterranean diet as represented by the Traditional Lebanese Mediterranean diet (LMD), to evaluate the association between the LMD and selected European Mediterranean diets (EMD), and to examine socio-demographic and lifestyle correlates of adherence to Mediterranean diet (MD) among Lebanese adults.

**Methods:**

Using nationally representative dietary intake data of Lebanese adults, an index to measure Adherence to the LMD was derived. The choice of foods/food groups used for calculating the LMD score was based on results of previous factor analyses conducted on the same dataset. These foods/food groups included fruits, vegetables, legumes, olive oil, burghol, dairy products, starchy vegetables, dried fruits and eggs. Using Pearson’s correlation and scores tertiles distributions agreement, the derived LMD index was compared to previously published EMD indexes from Greece, Spain, Italy, France and EPIC/Europe.

**Results:**

Fruits, vegetables and olive oil were common denominators to most MD scores. Food groups, specific to the LMD, included burghol, dried fruits, and eggs. The LMD score significantly correlated with the EMD scores, while being closest to the Italian (*r* = 0.56) and farthest from the French (*r* = 0.21). Percent agreement between scores’ tertile distributions and Kappa statistics confirmed these findings. Multivariate linear regressions showed that older age and higher educational levels were associated with increased adherence to all MDs studied.

**Conclusion:**

A novel LMD index was proposed to assess adherence to a Middle Eastern version of MD, complementing international efforts to characterize the MD and its association with disease risk.

## Introduction

The Mediterranean diet (MD) has been widely used to describe the dietary pattern that dominated in olive tree growing areas of the Mediterranean coastline [1]. Since 1975, when Keys [3] first used the term Mediterranean pattern, a plethora of studies investigated the health effects of this pattern. These studies conferred substantial evidence for a protective effect of the MD against the incidence of several non-communicable diseases (NCDs) including cardiovascular diseases (CVDs) and chronic degenerative diseases [2, 4–8]. A meta-analysis of prospective studies that investigated the effect of adherence to the Mediterranean dietary patterns on several health outcomes showed that increased adherence was associated with a significant reduction in overall mortality, CVD incidence or mortality, incidence or mortality from neoplasms and incidence of Parkinson’s and Alzheimer disease [6]. Another more recent meta-analysis showed that high adherence to the MD was consistently associated with reduced risk for stroke (RR  0.71, 95 % confidence interval [CI]  0.57–0.89), depression (RR  0.68, 95 % CI  0.54–0.86) and cognitive impairment (RR   0.60, 95 % CI   0.43–0.83) [9]. Furthermore, when different approaches to management of type 2 diabetes (T2D) were compared, a meta-analysis of 20 randomized clinical trials showed that adherence to MD leads to the largest improvement in glycemic control, weight loss and increase in HDL [10].

While the evidence for a protective effect of the MD has been consistent, the characteristics of this pattern are quite variable. While most definitions of the MD include fruits, vegetables and olive oil, significant differences exist in what relates to types of meat (processed meat, red meat and poultry), amount of fish, types and amount of fat or oil, types of dessert and sugar consumption, and amount and types of alcohol consumption [11]. Such discrepancies in what constitutes a particular MD could, to a large extent, depend on its country of origin. An investigation of the food habits in countries of the Mediterranean basin showed that food consumption varied not only between different Mediterranean countries but also within the same country [11]. These variations are influenced by various factors such as socio cultural, religious and economic determinants [1].

During the last few decades, many indexes have been proposed to assess adherence to various versions of the MD, with the majority of these indexes originating from countries in Europe, namely Italy, Spain, Greece, Crete and France [1]. Up to date, there exists no index for assessing adherence to a Middle Eastern version of the Mediterranean diet.

Lebanon is a small middle-income country on the Eastern shore of the Mediterranean Sea. According to earlier investigations by our group, Lebanon is host to four different dietary patterns: the Western, characterized by a high consumption of pizzas and pies, soda drinks, fast food sandwiches and sweets; the Prudent, consisting mostly of low fat milk and dairy products, whole bread, and breakfast cereals; the fish and alcohol which, as its name depicts, is characterized by a high consumption of fish and alcohol, in addition to the Traditional Lebanese pattern which encompasses high intakes of fruits, vegetables, legumes and olives [12]. In a case control study in Beirut, capital of Lebanon, a greater adherence to the Traditional Lebanese pattern was found to be associated with a lower risk of T2D among adults [13].

Using dietary intake data from a nationally representative sample of Lebanese adults, this study aims to propose an index for assessing adherence to a Middle Eastern version of the MD developed from the Traditional Lebanese dietary pattern and to assess the correlation between the proposed index and other indexes used to measure adherence to MDs in Europe. A secondary objective of this study is to examine the socio-demographic and lifestyle determinants of adherence to the various MDs including the Lebanese Mediterranean diet (LMD) in Lebanese adults.

### Methodology

The data for this study were drawn from the nation-wide nutrition and non-communicable diseases risk factors cross-sectional survey conducted in Lebanon between years 2008 and 2009. The sampling for this national survey was random, multistage (by governorate) and was based on the age-sex distribution of the Lebanese population [14]. Additional details about the sampling used in this study are described elsewhere [12]. Sample size calculations for the original study showed that 2,950 participants were needed to estimate an obesity prevalence of 30 % with 1.5 margin of error. The “30 %” figure was derived from an earlier national survey that took place in Lebanon in the year 1997 [15]. One adult, older than 20 years, was selected from each household using the household roster. Pregnant and lactating women and subjects with mental disabilities were excluded. A total of 3,178 subjects were approached and 2,613 accepted to participate in the survey (response rate 82.2 %). No information existed on subjects who refused to participate. For the purpose of this study, data for survey participants aged between 20 and 55 years were used (*n* = 2,048). The remaining 565 participants were excluded from this study as they were older than 55 years. In a one to one interview, study participants completed a brief socio-demographic and lifestyle questionnaire and a semi-quantitative food frequency questionnaire (FFQ). All the interviews were conducted by trained dietitians and took place at the participants’ homes. Further details about the survey and data collection procedures are described elsewhere [12].

Dietary intake of participants was assessed by a 61-item semi-quantitative FFQ that referred to food intake 1 year prior to the interview. For each food item listed on the FFQ, a standard portion size was indicated, and subjects were asked to record the frequency of consumption (open-ended) either per day, per week, per month, per year or never. Daily gram intakes of food items as well as the energy and nutrients composition of foods were computed using the food composition database of the nutritionist IV software [16] and the food composition table of Middle Eastern foods for local and traditional foods [17]. This FFQ was designed by a panel of nutritionists and included culture-specific dishes and recipes. It was tested on a convenient sample to check for clarity and cultural sensitivity. A few food items were renamed to appear in common language, for instance, meat pies were referred to as “kebbe”. Furthermore, the order of questions was changed to respect the cultural beliefs of the study population. FFQ items related to alcohol consumption were moved to the end, as study participants belonging to Muslim religion might be deterred by questions of the sort. The FFQ used in this study was administered by a trained dietitian and not self-completed. Such a modality of FFQ completion does not require a literate population, resulting in consistent interpretations, and higher response and completion rates, each of which may enhance the validity of the data [18]. Under and over-reporters were identified by the Goldberg method and using 2 standard deviations (SD) as the cutoff [19]. Of the study participants 67.7 % were normal-, 10.6 % were under- and 21.6 % were over-reporters. Characteristics of participants, excluding the under- and over-reporters, are presented in “Appendix 1”. Due to little differences between the two analyses (with and without under- and over-reporters), results pertaining to all subjects were presented in this paper.

The following variables were extracted from the original survey files and were included in the analysis: age (continuous in years), sex, income per month (<1.0 million L.L., 1–3 million L.L. and >3 million L.L.; 1,500 L.L. is equivalent to almost $1 USD), marital status (single including single, divorced, and widowed, and married including currently married or living with a partner), education (illiterate and primary, elementary, secondary, technical, and university and higher education), family history of obesity (a positive family history of obesity was indicated if the mother, father or both were reported as obese), and smoking (smokers including current smokers, non-smokers including non-smokers and past smokers). To assess physical activity among participants, the short version of the international physical activity questionnaire (IPAQ) was used. Three categories of physical activity were assigned based on METS-min per week (low: <600, moderate: at least 600 and high: at least 3,000) [20]. The weekly frequency of breakfast consumption, eating while watching television (TV), eating out, as well as daily frequency of snack consumption were reported as continuous variables.

### Derivation of the Lebanese Mediterranean diet (LMD) score

The derivation of a score to measure adherence to the LMD was based on the results of earlier investigations led by our group aiming to characterize the main dietary patterns in Lebanon. In these investigations, using factor analysis to derive dietary patterns, a Lebanese traditional pattern has consistently emerged as a common pattern. Out of 30 food groups/food items entered in the factor analysis, nine repeatedly loaded high on this pattern, including fruits, vegetables, legumes, olive oil, burghol (crushed whole wheat), milk and dairy products, starchy vegetables (including potato, corn and peas), dried fruits and eggs. Further details about the derivation of this pattern and its characterization are presented elsewhere [12, 13, 21]. The calculation of the LMD score in the current study was based on the number of portions of these nine foods/food groups consumed daily. Specifically, consumption data of each of these nine foods/food groups were divided into tertiles and a value of 1, 2, and 3 was assigned to the first, second and third tertiles of consumption, respectively. The Lebanese pattern score was then calculated, for each subject, as the sum of points received on the consumption of the nine foods/food groups. The higher the score, the greater was the adherence to LMD. The range for the possible scores was between 9 and 27 reflecting minimal and maximal adherence, respectively.

### Calculation of scores for the various European Mediterranean diet (EMD) indexes

In addition to the LMD, five other EMD scores were calculated. These scores belonged to previously defined Mediterranean diet indexes from four European countries of the western Mediterranean basin (Greece, Spain, Italy and France), as well as the EPIC (European Prospective Investigation Into Cancer)/Europe derived Mediterranean diet score.

The score for the Greek Mediterranean Diet Index (nMED) was calculated as per Panagiotakos et al. [22] in the context of the ATTICA study. This score was based on recommendations for consumption of 11 essential components of the Greek Mediterranean diet pyramid (non-refined cereals, fruits, vegetables, legumes, potatoes, fish, meat and meat products, poultry, full fat dairy products, as well as olive oil and alcohol intake) [23]. Individual rating from 0 to 5 or the reverse was assigned for increasing frequencies of consumption per month of foods according to their position in the Mediterranean diet pyramid. The reverse rating was assigned for foods at the middle or top of the pyramid. The score ranged from 0 to 55 with higher values indicating greater adherence to this diet [23].

The Italian Mediterranean Index (ITALIAN-MED) was developed by Agnoli et al. [24] for the Italian section of the EPIC cohort. It was based on 11 food items including 6 typical Mediterranean food items (pasta, Mediterranean vegetables, fruits, legumes, olive oil and fish), 4 non-Mediterranean food items (soft drinks, butter, red meat and potatoes) and alcohol. Individuals falling in the third tertile of consumption of typical Mediterranean food items received a score of 1 and 0 otherwise. For non-Mediterranean food items, a score of 1 was assigned for people falling in the first tertile of consumption and 0 otherwise. For alcohol, one point was assigned for those consuming up to 12 g per day, and 0 for abstainers or those consuming more than 12 g alcohol per day. Scores ranged between 0 and 11. Higher values of this diet score indicating greater adherence [24].

From Spain, the rMED index developed by Buckland et al. [25] was considered. A total of nine key components were included in the calculations of this index’s score. A value of 0, 1 and 2 was assigned to the first, second and third tertiles of intake, respectively, for the following food groups/foods: fruits (including nuts and seeds but excluding fruit juices), vegetables, legumes, cereals, fresh fish and olive oil. The scoring is reversed for meat and dairy products to positively score lower intakes. Alcohol is scored dichotomously giving a score of 2 for moderate consumers and 0 for above and below the sex-specific range. Possible scores ranged from 0 (minimal adherence) to 18 (maximum adherence) [25].

Gerber [26] developed a specific Mediterranean Diet Quality Index (Med-DQI) to describe the French Mediterranean diet, using dietary intake data of subjects belonging to a French Mediterranean area in Quebec, Canada,. The Med-DQI is a 7-item index based on the consumption of meats, olive oil, fish, cereals vegetables and fruits, in addition to saturated fatty acids (SFA) and cholesterol. Each food group or nutrient is assigned a score of 0, 1 and 2 on the basis of either recommended guidelines (such is the case for cholesterol and SFA) or by dividing the population’s consumption into tertiles (for meats, olive oil, fish, cereals vegetables and fruits). The scores (0, 1 and 2) were assigned in ascending order for cholesterol, SFA and meats and descending order for olive oil, fish, cereals, fruits and vegetables. The lower the Med-DQI, the healthier the diet was. The best Med-DQI had a score of 0 and the poorest, 14 [26]. For the purpose of comparison with other diets considered in the study, the MED-DQI scores were transformed using the following formula: [14-(MED-DQI)].

The Mediterranean diet scale (MDS), constructed to measure adherence to the Mediterranean diet in nine European countries (Denmark, France, Germany, Greece, Italy, the Netherlands, Spain, Sweden and the UK), was also computed and included 9 components. Individuals were assigned a score of 1 if their consumption of vegetables, legumes, fruits, cereals and fish was above the sex-specific median intake and 0 otherwise. The opposite scoring was given for meat and dairy. For alcohol, men consuming 10–50 g/day and women consuming 5–25 g/day were given a score of 1 and 0 otherwise. The ratio of monounsaturated fat (MUFA) and polyunsaturated fat (PUFA) to saturated fat (SFA) was also calculated. This scale ranged between 0 (minimal adherence) and 9 (maximal adherence) [27].

In this study, the dietary intake data collected by means of the FFQ were used to calculate the scores of the various indexes considered. Different groupings of foods and nutrients were run in accordance with the proposed method of score calculation for each index. “Appendix 2” provides a listing of the specific items in the FFQ that corresponded to the various components of each Mediterranean Diet Index.

### Statistical analysis

Socio-demographic and lifestyle characteristics of the study population were described using means ± standard deviations (SDs) and proportions for continuous and categorical variables, respectively. The normality of the scores was evaluated using the Kolmogorov–Smirnov and Shapiro–Wilk tests of normality. Both tests were significant, indicating that the scores did not follow a normal distribution. Spearman correlation coefficients were used to evaluate the association between the scores of the various MDs considered in this study. Study participants were grouped into tertiles using the scores of each MD. Exact and adjacent proportion agreements were calculated between the tertiles groups of the LMD scores and each of the MDs score. Cohen’s Kappa was also used to measure the strength of agreement between the LMD score and the various MD scores. Cohen’s Kappa was calculated based on the observed versus the expected agreement, and using the following formula: = (po−pe)/(1−pe), where po referred to probability of observed agreement and pe referred to expected agreements [28]. To assess the determinants of adherence to each of the MDs, multivariable logistic regression analysis was conducted with the score of each MD as outcome variable [first tertile (low adherence) vs second and third tertile (medium and high adherence)] and the socio-demographic and lifestyle characteristics as independent variables. The standard logistic regression modeling was used, where all the independent variables were entered into the regression equations at the same time. SPSS version 21.0 was used for all statistical analysis [29].

## Results

The socio-demographic and lifestyle characteristics of study participants are presented in Table 1 (*n* = 2,048). Overall, the study sample consisted of 45 % males (*n* = 923) and 55 % females (*n* = 1,125) with mean age 34.5 ± 10.0 and 34.8 ± 9.9 years, respectively. Seventy percent of study participants had a monthly income lower than 1,000,000 L.L. (equivalent to $667 USD), with males having significantly higher income than females. Compared to males, a lower percentage of females smoked cigarettes (*p* <0.05). Overall, almost half of the study population had a low level of physical activity (48.1 %). Average weekly breakfast consumption was about five times per week and was comparable between males and females. Compared to males, females reported lower frequencies of eating out and eating while watching TV (Table 1). Further details about the socio-demographic and lifestyle characteristics of the study population are presented elsewhere [12].

**Table 1:** Socio-demographic and lifestyle characteristics of study participants aged 20–55 years (*n* = 2,048)^a^ [20]

	Total (*n* = 2,048)	Males (*n* = 923)	Females (*n* = 1,125)	Significance^b^
Age (years)	34.7 ± 9.9	34.5 ± 10.0	34.8 ± 9.9	*p* > 0.05
Income per month (L.L.)				
<1 million	1,447 (70.8)	603 (65.5)	844 (75.2)	
1–3 million	501 (24.5)	264 (28.7)	237 (21.1)	
>3 million	96 (4.7)	54 (5.9)	42 (3.7)	χ^2^ = 23.36, *p* < 0.001
Marital status				
Single	861 (42.1)	462 (50.1)	399 (35.5)	
Married	1,185 (57.9)	460 (49.9)	725 (64.5)	χ^2^ = 44.4, *p* < 0.001
Education				
Illiterate and primary	269 (13.1)	130 (14.1)	139 (12.4)	
Elementary	514 (25.1)	243 (26.3)	271 (24.1)	
Secondary	346 (16.9)	143 (15.5)	203 (18.0)	
Technical	221 (10.8)	107 (11.6)	114 (10.1)	
University and higher	698 (34.1)	300 (32.5)	398 (35.4)	χ^2^ = 6.35, *p* > 0.05
Family history of obesity				
No	1,131 (55.5)	556 (60.6)	575 (51.3)	
Yes	907 (44.5)	362 (39.4)	545 (48.7)	χ^2^ = 17.39, *p* < 0.001
Smoking				
No	1,267 (61.9)	467 (50.6)	800 (71.1)	
Yes	781 (38.1)	456 (49.4)	325 (28.9)	χ^2^ = 90.45, *p* < 0.001
Physical activity level				
Low	841 (48.1)	434 (53.3)	407 (43.6)	
Moderate	485 (27.7)	211 (25.9)	274 (29.4)	
High	422 (24.1)	170 (20.9)	252 (27)	χ^2^ = 17.1, *p* < 0.001
Breakfast per week	4.7 ± 2.9	4.8 ± 2.9	4.6 ± 2.9	*p* > 0.05
Snack per day	1.5 ± 1.3	1.5 ± 1.4	1.5 ± 1.1	*p* > 0.05
Eating at TV per week	3.1 ± 3.2	3.5 ± 3.2	2.8 ± 3.1	*p* < 0.001
Eating out per week	1.6 ± 2.2	2.4 ± 2.5	1.0 ± 1.5	*p* < 0.001

^a^Categorical variables are expressed as *n* (%), continuous variables are expressed as mean ± (SDs)

^b^Significance is derived from independent *t* test for continuous variables and Chi-square test (χ^2^) for categorical variables

Table 2 shows the food groups, foods and nutrients used in the calculations of the scores of various MDs indexes considered in this study. Fruits, vegetables and olive oil were the three common denominators for Mediterranean indexes reported from Greece, Italy, Spain, France as well as Lebanon. Food groups and foods specific to the LMD included burghol, dried fruits and eggs. Fish was an integral component of all the MDs except the LMD. Alcohol was not used in the calculation of either the Lebanese or the French MD. Nutrients such as cholesterol and SFA were integrated in the calculations of the French MD while the ratio of the sum of MUFA and PUFA over SFA were used in that of the EPIC/Europe MD.

**Table 2:** Description of the various Mediterranean diets considered in this study and the mean scores of these diets among study participants (*n* = 2,048)

Food components	Lebanon^a,j^	Greece^b^	Italy^c^	Spain^d^	France^e,g^	EPIC/Europe^f^
Food groups						
Cereals	–	–	–	√ (+)	√ (+)	√ (+)
Non-refined cereal	–	√ (+)	–	–	–	–
Fruits	√ (+)	√ (+)	√ (+)	√ (+)	√ (+)	√ (+)
Dried fruits	√ (+)	–	–	–	–	–
Vegetables	√ (+)	√ (+)	√ (+)	√ (+)	√ (+)	√ (+)
Nuts and seeds	–	–	–	√ (+)	–	–
Legumes	√ (+)	√ (+)	√ (+)	√ (+)	–	√ (+)
Dairy products	√ (+)	√ (−)	–	√ (−)	–	√ (−)
Foods						
Burghol	√ (+)	–	–	–	–	–
Olive oil	√ (+)	√ (+)	√ (+)	√ (+)	√ (+)	–
Butter	–	–	√ (−)	–	–	–
Poultry	–	√ (−)	–	–	–	–
Red meat and/or meat products	–	√ (−)	√ (−)	√ (−)	√ (−)	√ (−)
Fish	–	√ (+)	√ (+)	√ (+)	√ (+)	√ (+)
Eggs	√ (+)	–	–	–	–	–
Pasta	–	–	√ (+)	–	–	–
Potatoes	√ (+)	√ (+)	√ (−)	–	–	–
Soft drinks	–	–	√ (−)	–	–	–
Alcohol	–	√ (−)	√ (*)	√ (*)	–	√ (*)
Nutrients						
Cholesterol	–	–	–	–	√ (−)	–
SFA (% energy)	–	–	–	–	√ (−)	–
(MUFA + PUFA)/SFA	–	–	–	–	–	√ (+)
Total number of components	9	11	11	9^h^	7^i^	9
Minimum possible score	9	0	0	0	0	0
Maximum possible score	27	55	11	18	14	9
Mean scores ± SD	17.38 ± 3.40	27.23 ± 4.65	3.56 ± 1.76	8.27 ± 2.49	6.20 ± 1.81	4.18 ± 1.49

(+) Was used when a higher score was assigned to a greater intake, (−) was used when a higher score was assigned to a lower intake, (*) was used when the highest score was assigned for moderate intake

^a^ Naja et al. [12], ^b^ Panagiotakos et al. [22], ^c^ Agnoli et al. [24], ^d^ Buckland et al. [25], ^e^ Gerber M. [26], ^f^ Trichopoulou A. et al. [27], ^g^ The scores for France MD were reversed using the following formula [14- (MED-DQI)] and the transformed scores were reported in this table, ^h^ The fruit group for this pattern included nuts and seeds, ^i^ For this pattern fruits and vegetables were combined into one group, ^j^ For the LMD, in lieu of “starchy vegetables” group, only “potato” group was added to the list of food components making up the LMD, as peas and corn were already included as part of the “vegetables” group

The associations between the various MD scores were evaluated using Spearman’s correlation (Table 3). Overall, 7 out of 15 correlation coefficients were in the acceptable range (between 0.3 and 0.49). Only the correlations between the French and Spanish MD scores and between the Italian MD and the LMD reached a “good” level of association and were equal or greater than 0.5 (*r* = 0.51 and 0.56; respectively). The LMD score had the highest correlation with scores of the Italian MD, followed by the Spanish (*r* = 0.30) and was least correlated with that of the French MD (*r* = 0.21). Among the MDs from Europe, the Spanish MD presented high correlations with the French and the Greek MDs (*r* = 0.51 and *r* = 0.44, respectively). The MD of Italy and France were least correlated (*r* = 0.06).

**Table 3:** Association between the various Mediterranean diets’ scores among study participants, as evaluated using Spearman correlation coefficients and their corresponding 95 % CI (*n* = 2,048)^a^

	Lebanon	Greece	Italy	Spain	France	EPIC/Europe
Lebanon	1	–	–	–	–	–
Greece	0.28 (0.24–0.32)	1	–	–	–	–
Italy	0.56 (0.53–0.59)	0.31 (0.27–0.35)	1	–	–	–
Spain	0.30 (0.26–0.34)	0.44(0.41–0.47)	0.14 (0.10–0.18)	1	–	–
France	0.21 (0.17–0.25)	0.30 (0.26–0.34)	0.06 (0.02–0.10)	0.51 (0.48–0.54)	1	–
EPIC/Europe	0.28 (0.24–0.32)	0.39 (0.35–0.43)	0.38 (0.34–0.42)	0.43 (0.39–0.46)	0.29 (0.25–0.33)	1

^a^All correlations are significant at *p* < 0.01 except for the correlation between the Italian and the French MD scores, the significance was *p* < 0.05

In order to examine the agreement between the LMD and the various EMDs, percent agreement between the scores’ tertile distributions and Kappa statistics were calculated (Table 4). In conformity with the correlation analysis, the highest agreement was observed between the LMD and the Italian MD with 53.17 and 92.38 % of subjects classified in the same and the same or adjacent tertiles, respectively. The Kappa statistics reached 0.49 indicating a moderate agreement. On the other hand, the least agreement was noted with the French MD whereby only 38.57 % of subjects belonged to the same tertiles. The Kappa statistics for the association between the LMD and the French MD was 0.19, reflecting slight agreement [28].

**Table 4:** Agreement of the scores tertiles’ distribution between the Lebanese pattern scores and the different Mediterranean diets’ scores in the study population (*n* = 2,048)

Patterns scores	% Agreement		Kappa
	Same tertiles	Same or adjacent tertiles	Weighted Kappa, 95 % CI
Greece	42.04	83.84	0.21 (0.18–0.24)
Italy	53.17	92.38	0.49 (0.45–0.52)
Spain	43.54	85.30	0.27 (0.24–0.30)
France	38.57	86.91	0.19 (0.16–0.22)
EPIC/Europe	43.41	83.45	0.22 (0.19–0.25)

The correlates of adherence to the various MDs considered in this study were examined (Table 5). Consistent across all MDs was the association with age, whereby the older the subjects, the higher was their adherence to the MD. Similarly a higher education level was also associated with higher scores for all of the MDs studied. For both Lebanon and Italy, compared to males, females were more likely to adhere to the MD. A healthier lifestyle, consisting of high levels of physical activity, no smoking and higher frequency of breakfast consumption, was associated with a higher adherence to the LMD. A higher frequency of snacking was significantly associated with the LMD, the Italian and the EPIC MDs, while no such association was observed with the Greek or the French MDs. A reversed relationship was noted between snacking and adherence to the Spanish MD. While “eating out” was associated with lower adherence to the LMD, Spanish and French MDs, it was positively associated with the scores of the Italian MD.

**Table 5:** Correlates of adherence to the various Mediterranean diets among study participants, using multivariable logistic regression (n = 2,048)^a^

	Lebanon	Greece	Italy	Spain	France	EPIC/Europe
Age (years)	1.04 (1.03–1.06)	1.02 (1.01–1.04)	1.02 (1.01–1.03)	1.06 (1.05–1.08)	1.04 (1.03–1.06)	1.04 (1.03–1.05)
Sex (female vs male)	1.67 (1.33–2.09)	1.19 (0.97–1.47)	2.51 (2.03–3.11)	0.74 (0.58–1.11)	0.82 (0.66–1.02)	0.81 (0.66–1.10)
Income per month (L.L.)						
<1 million	1	1	1	1	1	1
1–3 million	1.37 (1.08–1.75)	0.92 (0.74–1.15)	1.14 (0.91–1.43)	1.01 (0.80–1.27)	1.01 (0.81–1.28)	0.99 (0.80–1.23)
>3 million	1.34 (0.82–2.18)	0.93(0.60–1.42)	1.79(1.07–3.00)	0.81(0.51–1.30)	1.00 (0.63–1.59)	1.30 (0.83–2.04)
Marital status						
Single	1	1	1	1	1	1
Married	1.18 (0.92–1.51)	0.97 (0.77–1.22)	1.10 (0.87–1.39)	1.15 (0.90–1.46)	1.01 (0.80–1.30)	0.82 (0.65–1.03)
Education						
Illiterate and primary	1	1	1	1	1	1
Elementary	1.10 (0.78–1.55)	1.35 (0.98–1.86)	0.95 (0.68–1.32)	1.18 (0.85–1.65)	1.10 (0.78–1.54)	1.51 (1.10–2.07)
Secondary	1.23 (0.85–1.78)	1.49 (1.05–2.10)	0.94 (0.66–1.33)	1.69 (1.17–2.43)	1.29 (0.89–1.87)	1.69 (1.20–2.37)
Technical	1.56 (1.02–2.38)	1.72 (1.15–2.56)	1.01 (0.67–1.53)	2.55 (1.67–3.90)	1.56 (1.02–2.39)	2.06 (1.39–3.06)
University and higher	1.85 (1.29–2.64)	1.57 (1.13–2.19)	1.40 (0.99–1.96)	2.50 (1.80–3.48)	1.41 (1.00–1.99)	2.28 (1.64–3.15)
Family history of obesity						
No	1	1	1	1	1	1
Yes	1.03 (0.85–1.26)	0.88 (0.73–1.07)	0.90 (0.73–1.12)	1.02 (0.83–1.24)	1.08 (0.88–1.31)	0.79 (0.66–0.95)
Smoking						
No	1	1	1	1	1	1
Yes	0.78 (0.62–0.97)	0.97 (0.79–1.20)	0.92 (0.75–1.12)	0.71 (0.57–0.88)	0.76 (0.57–0.86)	1.15 (0.94–1.41)
Physical activity level						
Low	1	1	1	1	1	1
Moderate	0.95 (0.73–1.27)	1.18 (0.91–1.54)	1.00 (0.77–1.31)	1.10 (0.84–1.45)	0.72 (0.55–0.96)	0.83 (0.64–1.08)
High	1.62 (1.30–2.03)	1.38 (1.12–1.70)	1.34 (1.08–1.66)	1.31 (1.05–1.63)	1.02 (0.82–1.27)	1.14 (0.93–1.40)
Breakfast per week	1.08 (1.05–1.12)	1.00 (0.97–1.04)	0.98 (0.95–1.02)	1.03 (1.00–1.07)	1.05 (1.01–1.08)	0.97 (0.93–1.00)
Snack per day	1.26 (1.15–1.37)	1.07 (0.99–1.15)	1.26 (1.16–1.37)	0.89 (0.82–0.96)	0.97 (0.90–1.05)	1.11 (1.03–1.19)
Eating at TV per week	1.01 (0.98–1.04)	1.00 (0.97–1.03)	1.04 (1.01–1.07)	0.96 (0.93–0.99)	0.98 (0.95–1.01)	1.00 (0.97–1.03)
Eating out per week	0.93 (0.89–0.98)	0.99 (0.94–1.04)	1.11 (1.04–1.17)	0.90 (0.85–0.95)	0.89 (0.84–0.93)	1.06 (1.00–1.12)

^a^Values in this table reflect odds ratio (OR) and their respective 95 % confidence interval (CI)

## Discussion

This study is the first to propose an index for assessing adherence to a Middle Eastern version of the Mediterranean diet as represented by the Traditional Lebanese dietary pattern, which was previously shown to be protective against T2D [13]. The work undertaken in this study complements international efforts aiming to characterize the MD and to develop tools that can be used in identifying whole dietary patterns instead of single foods or nutrients [6]. Up to date, several Mediterranean adherence indexes have been proposed in the literature with all of these indexes originating from European countries, and none from the Eastern Mediterranean region. Given that the diet of the Eastern Mediterranean population has its own distinct features and particularities, the development of the LMD was the first step in exploring the Mediterranean diet in this region and in investigating its potential associations with disease risk. Findings may pave the way for holistic and culture-specific intervention strategies promoting the adoption of the LMD as a Mediterranean dietary pattern stemming from the region.

The results of this study showed that the LMD index was found to be the closest to the Italian Mediterranean index (*r* = 0.56) and the farthest from the French (*r* = 0.21). These findings highlight the inter-country discrepancies in what constitutes a MD and highlight the multiple variants and forms of this diet. In fact, even though a typical MD has been described and defined in the literature [3, 30], the Mediterranean-style diet is not one specific diet, but rather a collection of eating habits traditionally followed by people in the different countries bordering the Mediterranean Sea [6]. The inter-country variations in what constitutes a MD are influenced by numerous factors, including sociocultural, religious and economic determinants, to name a few [1].

In the present study and in agreement with the definition of the typical MD [3, 30], the proposed LMD index included fruits, vegetables and olive oil, which together represent a common denominator to the MD indexes reported from Greece, Italy, Spain and France. Food groups that were specific to the LMD index included burghol, dried fruits, eggs and dairy products. Burghol, or crushed whole wheat, is a characteristic of the traditional food heritage of Lebanon and several other Eastern Mediterranean countries including Turkey, Iraq, Iran, Syria and Egypt. [31]. Similarly, the diet in Lebanon as well as in other countries of the Eastern Mediterranean basin is characterized by the consumption of dried fruits such as raisins, figs, dates, apricots and apples, which are typically consumed as snacks or are used as raw material in bakery and confectionary industries [32]. Dairy products were also found to be a component of the LMD. Lebanon, as well as other countries of the Levant including Syria and Jordan, traditionally consumes large quantities of dairy products, which are typically consumed as fermented milk products such as yogurt, strained yogurt (labneh) and white cheese in brine [33]. Fish, which was an integral component of all the MD indexes, was not a constituent of the LMD. As shown by a previous food consumption survey conducted in Beirut, fish consumption in Lebanon is low (19.7 g/day among Lebanese adults), with 74 % of adult subjects consuming <2 servings of fish per week and 65 % <1 serving per week [34]. This feature is not unique to Lebanon as the annual consumption of fish and shellfish for countries of the region is also low, averaging 5.1 kg per caput per annum, which is less than half the average figure of 12.1 kg for world consumption [35]. This low intake of fish may be partially explained by the relatively high price index of seafood in these countries. Additionally, this low intake may be a reflection of social taboos or antipathies toward eating fish and seafood which appear to be common among the Muslim populations of the Mediterranean basin (but not of the Gulf) [36]. Certain Muslim sects have clear prohibitions against shellfish consumption as well as fish with no scales or fins, and in common customs and mentalities regular fish is not popular [36]. This social/ethnic aspect of fish consumption has been partly reflected by the observed association between fish consumption and alcohol drinking [12, 36]. In many countries of Muslim majority, renouncing alcoholic drink has become iconic in affirming Muslim identity and enforcing religious authority [36]. It is therefore not surprising that the LMD index was among the few Mediterranean indexes that did not include alcohol.

The results obtained in this study identified older age and higher educational levels as characteristics associated with increased adherence to all MD studied, including the LMD. The observed direct association between age and adherence to MD is in accordance with previous investigations. For instance, findings reported by Patino-Alonso et al. [37] showed that, in Spain, adherence was lower among individuals younger than 49 years of age compared to older individuals. A possible explanation may be that older adults tend to maintain traditional dietary habits as compared to younger generations who have greater exposure to new and “fashionable” food products [38]. The age gradient in adherence to the MD might also indicate a state of nutrition transition, from a traditional to a Western dietary pattern, a phenomenon that typically manifests itself in younger age groups as is currently experienced by many countries of the Mediterranean region [12, 34]. A few factors may be contributing to the progressive erosion of the Traditional diet in Lebanon, such as trade liberalization, climate change and lack of efficient national policy to promote and sustain production and consumption of foods characteristic to the country [39]. This transition may be further accentuated in Lebanon as well as other countries of the Eastern Mediterranean basin given the continuous and accrued social instability and political turmoil.

Our results also suggested that less educated subjects tend to have lower adherence to the MD, thereby suggesting a possible inverse socioeconomic gradient in healthy eating. These findings are in agreement with those reported by previous studies [38, 40] and are in line with the conclusions reported by Darmon and Drewnowski [41] indicating that higher-quality diets are mainly consumed by better educated individuals. Higher education levels may in fact be associated with higher nutrition knowledge, an essential precursor to healthy dietary habits [41]. Alternatively, the lower adherence to MD among subjects from lower education and socioeconomic backgrounds may, in part, reflect an economic obstacle to the adoption of a Mediterranean dietary style [42]. A study conducted in Spain showed that a MD is more expensive to follow than a Western dietary pattern, suggesting food cost as a prohibitive factor to adherence [43]. However, in the present study, income was not shown to be associated with adherence to MD.

In this study, adherence to the LMD as well as the Italian MD was shown to be higher among women compared to men. These findings are in agreement with those reported by Patino-Alonzo et al. [37] in Spain. The observed associations are in line with other studies reporting women as being more health-conscious and followers of dietary recommendations than men [34, 44].

Similarly, in the present study, and in agreement with several previous reports [45], a healthier lifestyle, consisting of high levels of physical activity, no smoking, higher frequency of breakfast consumption, and lower frequency of eating out was associated with adherence to the LMD. These results may be interpreted as a recurrent manifestation of the well-known clustering of behavioral risk factors, including smoking, physical inactivity and unbalanced diet [45], a clustering that is increasingly prevalent in countries undergoing the nutrition transition, including Lebanon and other countries of the Eastern Mediterranean region [46]. A distinctive feature of the nutrition transition is the erosion of the traditional lifestyle, the shift toward an increasingly energy dense dietary pattern, and the adoption sedentary mode of living. This departure from the tradition lifestyle, a lifestyle repeatedly shown to decrease the risk of NCDs and all-cause mortality [47], has been accompanied by an alarming increase in the prevalence and burden of obesity and other nutrition-related diseases in countries of the region [46]. In Lebanon, obesity prevalence rates among adults have increased from 17 % in 1997 to 28.2 % in 2009 [48], an increase that surpasses that reported from several developed countries during the past decade, including the United States of America (USA) [49]. The prevalence of cardiovascular diseases and T2D in countries of the region are also among the highest in the world [46].

The findings of this study ought to be considered in light of a few limitations. The dietary intake data used for this study were collected using a semi-quantitative FFQ. It is important to note that the food frequency questionnaire used in this study was not validated in our study population; however, in another study investigating dietary patterns and metabolic syndrome among Lebanese males using the same FFQ, significant correlations were found between dietary cholesterol intake and plasma cholesterol and LDL levels (Pearson correlation *r* = 0.3 and 0.2 for cholesterol and LDL, respectively) [50]. In addition, the shortcomings of the use of the FFQ for dietary assessment, such as measurement errors, reliance on memory, the limited number of food items included in the food list and the high proportion of low energy reporters, should be taken into account [18]. Nevertheless, studies have shown that the FFQ remains the most suitable dietary data collection tool in large epidemiological studies as it provides information on the habitual diet over longer periods of time and allows ranking of individuals according to food or nutrient intake [18, 51]. The dietary intake data was analyzed using a 1993 non-Lebanese nutrient database; however due to lack of resources in the setting of the study, it was not possible to obtain a more updated database. It remains noteworthy to mention that the use of such database will affect energy and nutrients intakes derivation but not the assessment of foods/food groups’ consumption. The potential change in food composition between years 1993 and 2008 may have resulted in attenuation of the observed associations. Furthermore, among the factors believed to affect dietary patterns is religion. However, given the political tension in the country among various religious parties, no data about religious beliefs were collected.

## Conclusion

 This study has proposed an index for assessing adherence to a Middle Eastern version of the MD, as represented by the Traditional Lebanese dietary pattern and compared the developed LMD index to selected indexes used to measure adherence to MD in Europe. The study showed that the score of the LMD index was significantly correlated with all the selected EMD indexes, while being the closest to the Italian MD. In addition to fruits, vegetables and olive oil, which were common denominators to indexes reported from Greece, Italy, Spain, France, the LMD index was distinctively characterized by the inclusion of burghol, dried fruits, and dairy products which are part of the traditional food heritage of Lebanon as well as other countries of the Eastern Mediterranean basin. The study showed that men, smokers, younger subjects, participants who were less physically active and those with lower education levels, were less likely to adhere to a Mediterranean dietary pattern, thus highlighting the need for stronger efforts of health promotion in these groups [38]. In this context, the positive association that was observed between physical activity and adherence to all MDs is noteworthy, suggesting that public health or preventive interventions must address diet and physical activity in conjunction [42]. Taken together, the study’s findings highlight the need for policies and strategies aiming at preserving and promoting the MD in Lebanon. These strategies may include nutrition education on the health benefits of the MD at the population level as well as the development of policies to preserve traditional healthy foods. It is important to consider that food consumption, as part of the MD, cannot be separated from other activities that constitute the “food event”, such as production, marketing, and social and cultural habits, which have been built throughout history [52]. Hence national and regional efforts should focus on the dissemination of the MD as a concept that embraces biodiversity, sustainability, quality, health and cultural heritage [52]. Through the dissemination of the MD as an “Intangible Cultural Heritage”, nutritionists, researchers, policy makers, academicians, media, producers and consumers ought to play an active role in preserving this diet [52], especially in light of evidence for its protective effects against T2D [13]. Population-wide community-based intervention programs that involve multisectorial partnerships and that are responsive to the sociocultural norms of the population must be put in place to promote and preserve this threatened heritage.

## Appendix 1: Results excluding under-reporters [*n* = 217 (10.6 %)] and over-reporters [*n* = 443 (21.6 %)]

See Tables 6, 7, 8, 9 and 10.

**Table 6:** Socio-demographic and lifestyle characteristics of study participants aged 20–55 years excluding under- and over-reporters^a,b^

	Total	Males	Females	Significance^c^
Age (years)	35.3 ± 9.9	35.4 ± 10.1	35.2 ± 9.8	*p* > 0.05
Income per month (L.L.)				
<1 million	990 (71.3)	395 (67.8)	595 (74.3)	
1–3 million	330 (23.8)	158 (47.9)	172 (21.5)	
>3 million	64 (4.6)	30 (5.1)	34 (4.2)	χ^2^ = 7.09, *p* < 0.05
Marital status				
Single	540 (38.9)	270 (46.2)	270 (33.7)	
Married	847 (61.1)	315 (53.8)	532 (66.3)	χ^2^ = 22.20, *p* < 0.001
Education				
Illiterate and primary	170 (12.2)	82 (14.0)	88 (11.0)	
Elementary	349 (25.1)	155 (26.5)	194 (24.2)	
Secondary	237 (17.1)	95 (16.2)	142 (17.7)	
Technical	142 (10.2)	66 (11.3)	76 (9.5)	
University and higher	490 (35.3)	187 (32.0)	303 (37.7)	χ^2^ = 8.02, *p* > 0.05
Family history of obesity				
No	616 (44.6)	233 (40.0)	383 (48.0)	
Yes	764 (55.4)	349 (60.0)	415 (52.0)	χ^2^ = 8.63, *p* < 0.05
Smoking				
No	876 (63.1)	303 (51.8)	573 (71.4)	
Yes	512 (36.9)	282 (48.2)	230 (28.6)	χ^2^ = 55.63, *p* < 0.001
Physical activity level				
Low	574 (41.1)	275 (47.0)	299 (37.3)	
Moderate	255 (18.4)	106(18.1)	149 (18.6)	
High	558 (40.2)	204 (34.9)	354 (44.1)	χ^2^ = 14.99, *p* < 0.05
Breakfast per week	4.7 ± 2.9	4.8 ± 2.9	4.6 ± 2.8	*p* > 0.05
Snack per day	1.4 ± 1.2	1.4 ± 1.3	1.5 ± 1.1	*p* > 0.05
Eating at TV per week	3.0 ± 3.1	3.5 ± 3.2	2.7 ± 3.1	*p* < 0.001
Eating out per week	1.5 ± 2.1	2.1 ± 2.4	0.97 ± 1.6	*p* < 0.001

^a^Under-reporters [*n* = 217 (10.6 %)] and over-reporters [*n* = 443 (21.6 %)] (percentages of under- and over-reporters are calculated based on the original *n* = 2,048)

^b^Categorical variables are expressed as *n* (%), continuous variables are expressed as Mean ± (SDs)

^c^Significance is derived from independent *t* test for continuous variables and Chi square test (χ^2^) for categorical variables

**Table 7:** Description of the various Mediterranean diets considered in this study and the mean scores of these diets among study participants, excluding under- and over-reporters (*n* = 1,388)

Food components	Lebanon^a,j^	Greece^b^	Italy^c^	Spain^d^	France^e.g^	EPIC/Europe^f^
Food groups						
Cereals	–	–	–	√ (+)	√ (+)	√ (+)
Non-refined cereal	–	√ (+)	–	–	–	–
Fruits	√ (+)	√ (+)	√ (+)	√ (+)	√ (+)	√ (+)
Dried fruits	√ (+)	–	–	–	–	–
Vegetables	√ (+)	√ (+)	√ (+)	√ (+)	√ (+)	√ (+)
Nuts and seeds	–	–	–	√ (+)	–	–
Legumes	√ (+)	√ (+)	√ (+)	√ (+)	–	√ (+)
Dairy products	√ (+)	√ (−)	–	√ (−)	–	√ (−)
Foods						
Burghol	√ (+)	–	–	–	–	–
Olive oil	√ (+)	√ (+)	√ (+)	√ (+)	√ (+)	–
Butter	–	–	√ (−)	–	–	–
Poultry	–	√ (−)	–	–	–	–
Red meat and/or meat products	–	√ (−)	√ (−)	√ (−)	√ (−)	√ (−)
Fish	–	√ (+)	√ (+)	√ (+)	√ (+)	√ (+)
Eggs	√ (+)	–	–	–	–	–
Pasta	–	–	√ (+)	–	–	–
Potatoes	√ (+)	√ (+)	√ (−)	–	–	–
Soft drinks	–	–	√ (−)	–	–	–
Alcohol	–	√ (–)	√ (*)	√ (*)	–	√ (*)
Nutrients						
Cholesterol	–	–	–	–	√ (−)	–
SFA (% energy)	–	–	–	–	√ (−)	–
(MUFA + PUFA)/SFA	–	–	–	–	–	√ (+)
Total number of components	9	11	11	9^h^	7^i^	9
Minimum possible score	9	0	0	0	0	0
Maximum possible score	27	55	11	18	14	9
Mean scores ± SD	17.27 ± 3.22	27.3 ± 4.60	3.50 ± 1.63	8.45 ± 2.43	6.37 ± 1.76	4.16 ± 1.52

(+) Was used when a higher score was assigned to a greater intake, (−) was used when a higher score was assigned to a lower intake, (*) was used when the highest score was assigned for moderate intake

^a^Naja et al. [12], ^b^ Panagiotakos et al. [22], ^c^ Agnoli et al. [24], ^d^ Buckland et al. [25], ^e^ Gerber M. [26], ^f^ Trichopoulou A. et al. [27], ^g^ The scores for France MD were reversed using the following formula [14-(MED-DQI)] and the transformed scores were reported in this table, ^h^ The fruit group for this pattern included nuts and seeds, ^i^ For this pattern fruits and vegetables were combined into one group, ^j^ For the LMD, in lieu of “starchy vegetables” group, only “potato” group was added to the list of food components making up the LMD, as peas and corn were already included as part of the “vegetables” group

**Table 8:** Association between the various Mediterranean diets’ scores among study participants, as evaluated using Spearman correlation coefficients and their corresponding 95 % CI, excluding under- and over-reporters (*n* = 1,388)^a^

	Lebanon	Greece	Italy	Spain	France	EPIC/Europe
Lebanon	1	–	–	–	–	–
Greece	0.31 (0.27–0.35)	1	–	–	–	–
Italy	0.51 (0.48–0.54)	0.38 (0.34–0.42)	1	–	–	–
Spain	0.38 (0.34–0.42)	0.44 (0.41–0.47)	0.25 (0.21–0.29)	1	–	–
France	0.28 (0.24–0.32)	0.29 (0.25–0.33)	0.15 (0.11–0.19)	0.51 (0.48–0.54)	1	–
EPIC/Europe	0.27 (0.23–0.31)	0.40 (0.36–0.43)	0.38 (0.34–0.42)	0.48 (0.45–0.51)	0.31 (0.27–0.35)	1

^a^All correlations are significant at *p* < 0.01

**Table 9:** Agreement of the scores tertiles’ distribution between the Lebanese pattern scores and the different Mediterranean diets’ scores in the study population, excluding under- and over-reporters (*n* = 1,388)

Patterns scores	% Agreement		Kappa
	Same tertiles	Same or adjacent tertiles	Weighted Kappa (95 % CI)
Greece	43.08	85.23	0.24 (0.20–0.28)
Italy	51.44	92.58	0.45 (0.41–0.50)
Spain	46.33	87.54	0.34 (0.29–0.38)
France	39.27	89.34	0.25 (0.21–0.29)
EPIC/Europe	44.60	83.57	0.23 (0.19–0.26)

**Table 10:** Multivariate logistic regression for the correlates of adherence to the various Mediterranean diets among study participants excluding under- and over-reporters (*n* = 1,388)^a^

	Lebanon	Greece	Italy	Spain	France	EPIC/Europe
Age (years)	1.06 (1.04–1.07)	1.02 (1.01–1.04)	1.01 (1.00–1.03)	1.07 (1.05–1.09)	1.06 (1.04–1.08)	1.04 (1.03–1.06)
Sex (female vs male)	1.53 (1.17–2.01)	1.20 (0.93–1.54)	2.52 (1.65–3.26)	0.62 (0.48–0.81)	0.78 (0.60–1.03)	0.77 (0.60–0.98)
Income per month (L.L.)						
<1 million	1.00	1.00	1.00	1.00	1.00	1.00
1–3 million	1.34 (0.99–1.81)	0.91 (0.69–1.20)	1.03 (0.79–1.36)	0.94 (0.70–1.25)	0.98 (0.73–1.32)	1.11 (0.86–1.45)
>3 million	1.14 (0.63–2.05)	0.75 (0.44–1.29)	1.60 (0.88–2.89)	0.71 (0.40–1.25)	1.00 (0.56–1.80)	1.45 (0.83–2.53)
Marital status						
Single	1.00	1.00	1.00	1.00	1.00	1.00
Married	1.28 (0.95–1.72)	0.92 (0.69–1.21)	0.74 (0.71–1.25)	1.11 (0.82–1.50)	1.04 (0.76–1.41)	0.77 (0.58–1.01)
Education						
Illiterate and primary	1.00	1.00	1.00	1.00	1.00	1.00
Elementary	1.14 (0.75–1.74)	1.13 (0.76–1.67)	1.05 (0.71–1.56)	1.17 (0.77–1.78)	1.19 (0.77–1.84)	1.29 (0.87–1.900)
Secondary	1.36 (0.86–2.14)	1.34 (0.87–2.06)	1.22 (0.79–1.86)	1.52 (0.96–2.40)	1.41 (0.88–2.28)	1.42 (0.93–2.16)
Technical	1.66 (0.98–2.82)	1.34 (0.81–2.21)	1.23 (0.75–2.03)	2.21 (1.29–3.78)	1.57 (0.91–2.70)	1.82 (1.11–2.97)
University and higher	2.20 (1.41–3.42)	1.42 (0.94–2.14)	1.76 (1.17–2.65)	2.58 (1.65–4.03)	1.68 (1.07–2.63)	2.05 (1.37–3.06)
Family history of obesity						
No	1.00	1.00	1.00	1.00	1.00	1.00
Yes	1.07 (0.84–1.38)	0.80 (0.64–1.01)	0.94 (0.74–1.18)	0.97 (0.75–1.23)	0.96 (0.75–1.24)	0.83 (0.66–1.04)
Smoking						
No	1.00	1.00	1.00	1.00	1.00	1.00
Yes	0.70 (0.53–0.92)	0.91 (0.71–1.18)	0.76 (0.59–0.98)	0.69 (0.53–0.91)	0.73 (0.55–0.97)	1.21 (0.94–1.55)
Physical activity level						
Low	1.00	1.00	1.00	1.00	1.00	1.00
Moderate	0.98 (0.70–1.37)	1.19 (0.86–1.64)	1.02 (0.74–1.41)	1.16 (0.83–1.64)	0.79 (0.56–1.11)	0.78 (0.57–1.06)
High	1.67 (1.26–2.20)	1.27 (0.98–1.65)	1.56 (1.20–2.03)	1.10 (0.84–1.45)	0.96 (0.73–1.27)	1.07 (0.83–1.37)
Breakfast per week	1.04 (1.03–1.12)	1.02 (0.98–1.06)	0.96 (0.92–1.00)	1.03 (0.99–1.08)	1.03 (0.99–1.07)	0.97 (0.93–1.01)
Snack per day	1.22 (1.10–1.37)	1.15 (1.04–1.27)	1.18 (1.07–1.31)	0.94 (0.85–1.04)	1.03 (0.93–1.15)	1.11 (1.00–1.22**)**
Eating at TV per week	0.97 (0.94–1.01)	1.00 (0.97–1.04)	1.03 (0.99–1.07)	0.98 (0.94–1.01)	0.96 (0.92–1.00)	0.99 (0.95–1.03)
Eating out per week	0.93 (0.87–0.99)	0.99 (0.93–1.05)	1.07 (1.00–1.14)	0.92 (0.86–0.98)	0.90 (0.84–0.96)	1.06 (0.99–1.13)

^a^Values in this table reflect odds ratio (OR) and their respective 95 % confidence interval (CI)

## Appendix 2

See Table 11.

**Table 11:** List of specific items in the FFQ that correspond to the components of the various Mediterranean diet indexes ^a^

	Items in the FFQ
Food groups	
Cereals^a^	Rice; pasta; bread
Non-refined cereals	Burghol; whole bread
Fruits^b^	Citrus (e.g., orange); deep yellow or orange (e.g., peach, apricots); strawberry; grapes; banana; apple; fresh fruit juice
Dried fruits	Dried fruits (raisins, dates, apricots, figs, apples)
Vegetables	Salad greens (e.g., lettuce, celery, green peppers and cucumbers); dark or deep yellow vegetables (e.g., spinach, chicory, carrots); tomatoes (fresh and cooked); squash and eggplant; cruciferous vegetables (e.g., cauliflower, cabbage and broccoli); corn and peas
Nuts and seeds	Nuts and seeds (e.g., peanuts, almonds and sunflower seeds)
Legumes	Legumes (e.g., lentils, broad beans and chickpeas)
Dairy products	Whole fat milk; fat free and low fat milk; whole fat yogurt; fat free and low fat yogurt; labneh (strained yogurt); whole fat cheese; fat free and low fat cheese
Foods	
Burghol^c^	Burghol
Olive oil	Olive oil
Butter	Butter; ghee
Poultry	Poultry
Red meat and/or meat products	Red meat; organ meat (e.g., liver, kidney and brain); luncheon meats (e.g., mortadella, jambon and salami, turkey); sausages (e.g., makanek and hot dogs)
Fish	Fish (including canned tuna)
Eggs	Eggs
Pasta	Pasta
Potatoes	Potato (baked/boiled/mashed); fried potato
Soft drinks	Regular soft drinks; diet soft drinks
Alcohol	Liquor (e.g., whiskey, vodka, gin and rum); wine; beer

Items in the FFQ that corresponded to the various foods/food groups are separated by semi-colons

^a^For the Italian MD, pasta was considered a separate item while for the Spanish, French and EPIC/Europe MDs, “pasta” was part of the “cereals” group

^b^The food group “fruits” for the LMD excluded dried fruits as the latter was considered as a separate group

^c^For the LMD, “burghol” was a separate item and it was added to the “non-refined cereals” group for the Greek MD
